# Primary Cilia: The Chemical Antenna Regulating Human Adipose-Derived Stem Cell Osteogenesis

**DOI:** 10.1371/journal.pone.0062554

**Published:** 2013-05-17

**Authors:** Josephine C. Bodle, Candace D. Rubenstein, Michelle E. Phillips, Susan H. Bernacki, Jie Qi, Albert J. Banes, Elizabeth G. Loboa

**Affiliations:** 1 Joint Department of Biomedical Engineering, University of North Carolina at Chapel Hill and North Carolina State University, Raleigh, North Carolina, United States of America; 2 Flexcell International Corporation, Hillsborough, North Carolina, United States of America; 3 Department of Materials Science and Engineering, North Carolina State University, Raleigh North Carolina, United States of America; University of Minnesota Medical School, United States of America

## Abstract

Adipose-derived stem cells (ASC) are multipotent stem cells that show great potential as a cell source for osteogenic tissue replacements and it is critical to understand the underlying mechanisms of lineage specification. Here we explore the role of primary cilia in human ASC (hASC) differentiation. This study focuses on the chemosensitivity of the primary cilium and the action of its associated proteins: polycystin-1 (PC1), polycystin-2 (PC2) and intraflagellar transport protein-88 (IFT88), in hASC osteogenesis. To elucidate cilia-mediated mechanisms of hASC differentiation, siRNA knockdown of PC1, PC2 and IFT88 was performed to disrupt cilia-associated protein function. Immunostaining of the primary cilium structure indicated phenotypic-dependent changes in cilia morphology. hASC cultured in osteogenic differentiation media yielded cilia of a more elongated conformation than those cultured in expansion media, indicating cilia-sensitivity to the chemical environment and a relationship between the cilium structure and phenotypic determination. Abrogation of PC1, PC2 and IFT88 effected changes in both hASC proliferation and differentiation activity, as measured through proliferative activity, expression of osteogenic gene markers, calcium accretion and endogenous alkaline phosphatase activity. Results indicated that IFT88 may be an early mediator of the hASC differentiation process with its knockdown increasing hASC proliferation and decreasing Runx2, alkaline phosphatase and BMP-2 mRNA expression. PC1 and PC2 knockdown affected later osteogenic gene and end-product expression. PC1 knockdown resulted in downregulation of alkaline phosphatase and osteocalcin gene expression, diminished calcium accretion and reduced alkaline phosphatase enzymatic activity. Taken together our results indicate that the structure of the primary cilium is intimately associated with the process of hASC osteogenic differentiation and that its associated proteins are critical players in this process. Elucidating the dynamic role of the primary cilium and its associated proteins will help advance the application of hASC in generating autologous tissue engineered therapies in critical defect bone injuries.

## Introduction

**Figure 1:**
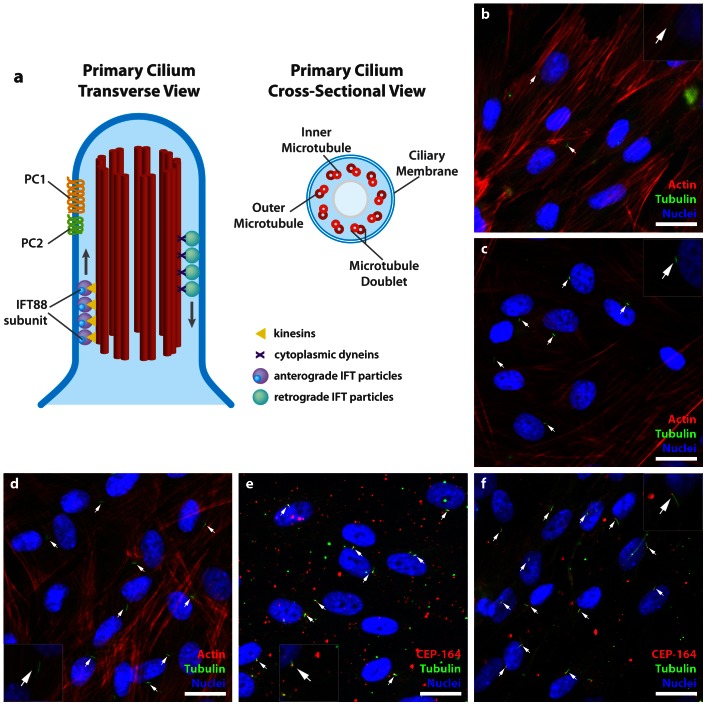
Primary cilia on hASC. (a) The non-motile primary cilium axoneme is composed of 9 pairs of peripheral microtubule doublets arranged concentrically in 9+0 configuration, devoid of a central tubulin pair. (b–f) Primary cilia appear as a dense cluster of acetylated tubulin protruding from the apical surface of hASC in confluent culture. (b) Few cilia are observed 24 hours after culture in complete growth expansion media (CGM) in subconfluent culture. They are observed at day 3 (c) undifferentiated in CGM, (d) differentiated in osteogenic differentiation medium (ODM) and at day 12 in (e) undifferentiated in CGM and (f) differentiated in ODM. Cilia are identified with anti-acetylated tubulin (green) or in combination with CEP164 (red), actin identified with phalloidin (red) and nuclei are identified with DAPI staining (blue). Scale bar = 25 µm.

The non-motile primary cilium is an organelle composed of tubulin, which projects from the centrosome and is located at the apical cell surface. The cilium axoneme consists of a set of nine peripheral microtubule doublets arranged in a 9+0 non-motile primary cilium configuration **(** **Fig. 1a** **)**. This is in contrast to the (9+2) motile cilium configuration, which has a central tubulin pair surrounded by nine tubulin doublets. Discovered over a century ago, non-motile primary cilia were largely considered vestigial organelles, despite their prevalence on a variety of cell types [1]. More recently, they have been implicated as critically important chemo- and mechanoresponsive cell surface structures, hinting at their role in functional phenotypic maintenance in a variety of mammalian cell types [2], [3]. Assembly of the primary cilium is cell cycle dependent and most mammalian cell types express primary cilia at some point during the cell cycle [3]. The scope of the primary cilium's function remains largely elusive, though evidence suggests that its function is complex. It acts as an important site for intracellular signaling [4] and detects external chemical and mechanical changes in the extracellular environment [5]. Emerging evidence from our group and others suggests that, in addition to tissue homeostasis, they may also be involved in signaling stem cell lineage commitment in both embryonic as well as in adult stem cells, both *in vivo* and *in vitro* [5]–[7].

Human adipose-derived stem cells (hASC) are a promising autologous cell source for tissue-engineered replacement therapies [8], [9]. Due to their relative abundance and multipotent capacity to differentiate into a variety of mesenchymal lineages, hASC have demonstrated potential for a broad range of therapeutic applications [8]–[11]. However, a majority of the mechanisms controlling these differentiation processes have largely remained elusive. We hypothesize the primary cilium and its associated proteins may contribute to these processes.

Primary cilia have been most thoroughly studied in kidney epithelial cells under the autosomal polycystic kidney disease model. Loss of the cilium and its associated proteins polycystin-1 (PC1) and polycystin-2 (PC2) in kidney epithelial cells is associated with the loss of epithelial cell mechanosensitivity, which leads to development of cysts and deregulation of tissue morphogenesis [12], [13]. PC1 and PC2 colocalize to the primary cilium **(** **Fig. 1a** **)** and are thought to have a large component of their functional activity at the site of the cilium [14]. However, it is important to note both PC1 and PC2 localize to other subcellular compartments as well. PC1 is observed at the apical cell surface and at cell-cell adhesion junctions in a variety of ductal and epithelial cell types [15], [16]. PC2 is found in the plasma membrane and in the endoplasmic reticulum of kidney epithelial cells [14].

In addition to PC1 and PC2, intraflagellar transport protein-88 (IFT88), also known as polaris, is another critical cilia-associated protein required for proper cilia structure and function **(** **Fig. 1a** **)**. Disease models incorporating IFT88 knockout result in a cystic kidney phenotype similar to that observed in autosomal polycystic kidney disease [17]. This is likely due to the fact that IFT88 is responsible for transporting proteins into the cilium axoneme, building the cilium microtubule structure, and transporting functional cilia proteins into the cilium. Due to this critical activity, disrupted IFT88 expression results in a shortened ciliary axoneme [17].

IFT88 activity in primary cilia has been of particular interest in bone cell cilia-mediated mechanosensing. Transient knockdown of IFT88 has been reported to reduce the frequency of primary cilia observed on osteoblastic cell lines. Further, IFT88 knockdown leads to reduced expression of osteogenic gene markers known to be upregulated with fluid shear stimulation, indicating a suppression of functional osteogenic phenotype [18].

In this study, we investigate the role of the primary cilium in hASC differentiation. Human ASC are excellent cell source candidates for musculoskeletal tissue engineering and regenerative medicine applications; however, characterizing the mechanisms of hASC differentiation is a barrier to implementing their clinical use. We hypothesize that primary cilia are present on hASC and that their associated proteins PC1, PC2 and IFT88 dynamically control hASC differentiation potential. Based on previous work by our group, as well as others, hASC are known to readily differentiate into an osteogenic cell phenotype. They upregulate osteogenic gene markers, and proteins, as well as accreting calcium and exhibiting changes in alkaline phosphatase activity, in response to a variety of osteogenic induction factors [10], [19]–[21]. The aim of this study is to elucidate the primary cilia-mediated mechanisms of the hASC differentiation process, specifically exploring the action of PC1, PC2 and IFT88.

## Materials and Methods

### Cell Isolation and Propagation

Human adipose-derived stem cells (hASC) are obtained from waste adipose tissue derived from anonymous patients undergoing elective abdominoplasty surgeries (IRB exemption protocol #10-0201), at University of North Carolina hospitals (Chapel Hill, NC). All data were analyzed anonymously and the only patient information obtained included age, sex and ethnicity. The Office of Human Research at UNC hospitals deemed that this study does not constitute human research, “as defined under federal regulations [45 CFR 46.102 (d or f) and 21 CFR 56.102(c)(e)(l)] and does not require IRB approval.” Isolation of hASC was performed according to previously established protocols from our lab [22], adapted from methods initially reported by Zuk et al. [10]. Once isolated, hASC obtained from approximately 50 g of tissue were allowed to propagate in culture in complete growth medium (CGM) until ∼80% confluency (or up to two weeks). CGM contained Eagle's Minimum Essential Medium, alpha-modified supplemented with 10% fetal bovine serum, 2 mM L-glutamine, 100 units/ml penicillin and 100 µg/ml streptomycin. The hASC were then trypsinized and frozen down at passage 0 (p0). All hASC used in these experiments were passage 4 or lower, and were derived from three female donors (ages 47–55 years).

### Osteogenic and Adipogenic Differentiation

To characterize the growth and differentiation potential of each cell line isolated per donor, p0 cells were grown in complete growth medium (CGM), adipogenic differentiation medium (ADM) and osteogenic differentiation medium (ODM). ADM contained the aforementioned CGM supplemented with 1 µM dexamethasone, 5 µg/ml insulin, 100 µM indomethacin and 500 µM isobutylmethylxanthine. ODM contained complete growth medium with the addition of 50 µM ascorbic acid, 0.1 µM dexamethasone, and 10 mM β -glycerolphosphate.

To confirm multipotency, p0 hASC were grown in each medium condition for 14 days to evaluate adipogenic and osteogenic differentiation potential. Cells were seeded in 6-well plates (5×10^4^ cells/well) and 12-well plates (2×10^4^ cells/well) and were grown to 90–100% confluency in CGM (1–3 days), then transferred to ODM or ADM (14 days) for osteogenic or adipogenic differentiation induction, respectively. Evidence of differentiation was visualized using Alizarin Red S for calcium accumulation in osteogenesis and Oil Red O for lipid droplet accumulation in adipogenesis.

### siRNA Knockdown

hASC at ∼60% confluency were transfected with Lipofectamine 2000® (Invitrogen, Carlsbad, CA) using siRNA knockdown for PC1, PC2 and IFT88. A ratio of 24 pmol siRNA to 1.2 µL lipofectamine 2000® per sample in a 12-well plate were prepared according to manufacturer's protocol. hASC were transfected for 4–5 hours exposure to siRNA-lipofectamine solution in serum-free Opti-MEM-I transfection media. Cells were placed in complete growth media following initial transfection and subsequently placed in treatment media (CGM, ODM, ADM) 24 hours later. Serial knockdown was applied at Day 0 and 7 for experiments lasting longer than 7 days.

To assess initial knockdown efficiency to optimize knockdown protocol, RNA was collected from duplicate samples of hASC transfected with either PC1 siRNA or Scramble-A control siRNA at 24, 48 and 72 hours after transfection. Real-time PCR using Taqman® was used to evaluate the relative mRNA expression levels between time-dependent controls and polycystin-1 knockdown samples. Following optimization with PC1, qRT-PCR was used to further evaluate mRNA expression of PC1, PC2 and IFT88 following knockdown.

### Immunostaining

hASC were fixed in 10% formalin for 15 minutes. Following fixation, cells were blocked with a 0.2% Triton X-100/5.0% BSA stock solution for 40 minutes in 12-well plates on a shaker table. Following blocking, the coverslips were incubated in the primary antibody solution in humidified chambers. Primary antibody solutions contained mouse monoclonal acetylated α-tubulin antibody (diluted 1∶100) (Sigma), CEP164 (1∶50) (Santa Cruz) rabbit PC1 (diluted 1∶50) (Santa Cruz), rabbit PC2 (diluted 1∶50) (SantaCruz) or goat IFT88 primary antibody (1∶50) (Abcam), with 0.2% Triton X-100, and 0.5% BSA in PBS. The cells were then incubated in the chambers overnight at 4°C.

Samples were rinsed three times in PBS for five minutes each on a shaker table and then incubated in the secondary antibody (1∶250), phalloidin 594 (Molecular Probes) (1∶500) and DAPI (1∶500) stain solutions. A chicken anti-mouse Alexa Fluor 488 (Molecular Probes) secondary (Molecular Probes) was used against the mouse acetylated α-tubulin primary, donkey anti-goat Alexa Fluor 594 secondary (Molecular Probes) was used against the goat IFT88 and CEP164 primaries and goat anti-rabbit Alexa Fluor 594 secondary (Molecular Probes) was used against PC1 and PC1 primaries. The samples were incubated for three hours at room temperature. Following the secondary incubation, the samples were placed in PBS for the final three rinses and excess liquid was blotted on a kimwipe in preparation for mounting. Prolong Gold Mounting Media (Molecular Probes) was used to mount the samples and slides were allowed to dry in the dark for 24 hours prior to imaging.

### Phase Contrast and Fluorescence Microscopy

Phase contrast microscopy was used to monitor hASC viability and to visualize cell morphology throughout the duration of differentiation. Fluorescence microscopy was used in conjunction with immunohistochemistry to visualize cytoskeletal organization, cell surface markers, primary cilia conformation, PC1, PC2 and IFT88 localization, cell-cell junctions and matrix deposition.

Samples were imaged on a Leica DM5500B Fluorescent Microscope using the compatible LAS-AF software. Three images were taken for each section: DAPI (blue), Alexa 488 (green), and Alexa 594 (red). They were used for nuclei visualization, cilia visualization, and actin/CEP164/PC1/PC2/IFT88 visualization respectively. Optimized exposure, gain, and intensity experiment settings were determined by using the quick LUT function of the LAS-AF program. Images were taken at 40× magnification, 500–1000 ms exposure time (depending on filter), and a lamp intensity of 4.

### Cellular Proliferation

To measure proliferative activity, DNA content was measured at days 0, 3, 5, 7 and 10. Cell lysates were digested overnight at 60°C in 2.5 units/mL papain from papaya latex in PBS with 5 mM ethylenediaminetetra-acetic acid and 5 mM cysteine hydrochloride. Hoechst 33258 (Molecular Probes), a DNA-binding fluorescent dye, was applied to samples at 0.1 µg/mL in a black 96-well microplate. Fluorescence was measured at an excitation wavelength of 352 nm and an emission wavelength of 461 nm on a microplate reader (TECAN GENios, Männedorf, Switzerland). A DNA standard curve was generated using DNA derived from calf thymus (Sigma) and DNA concentration was calculated as a function of fluorescence.

### RNA Extraction and Gene Expression Analysis

Quantitative RT-PCR analysis was used to determine relative mRNA expression levels of lineage specific gene markers for osteogenic gene markers. RNA cell lysate samples collected at days 0, 3, 10 and 14 were run through a Qiashredder column (Qiagen, Valencia, CA) at 15,000 g for 2 minutes to homogenize samples. Total RNA was extracted using the RNeasy Mini Kit (Qiagen, Valencia, CA). RNA concentrations were measured using a NanoDrop spectrophotometer (Thermo Scientific, Wilmington, DE). To synthesize first strand cDNA, 110–600 ng of RNA in a 21 µL reverse transcriptase (RT) reaction was used with the Superscript III RT with oligo(dT) primers kit (Invitrogen, Carlsbad, CA). Taqman Gene expression assays with pre-determined primer-probe sets were used to amplify diluted (1∶1) cDNA (Assays-on-Demand, Applied Biosystems, Foster City, CA). All gene expression profiles were normalized to Glyceraldehyde-3-phosphate dehydrogenase (GAPDH) (Assay HS99999905_M1) in an ABI Prism 7000 system.

### Histological Staining

After 14 days of culture in osteogenic differentiation medium, or complete growth medium, hASC were fixed in 10% formalin for 15 minutes and rinsed 2 times with deionized water. They were stained with a 2% (w/v) Alizarin Red S solution to characterize calcium accretion and degree of hASC osteogenic differentiation. After exposure to stain for 3 minutes, wells were rinsed 4 times and stains were qualitatively evaluated and visualized using a dissecting microscope (Leica Microsystems, Buffalo Grove, IL).

### Calcium Accretion

Total calcium concentration was quantified on day 14 using an absorbance assay. Briefly, cells were rinsed with PBS, and digested in 0.5 N HCl overnight. Samples were centrifuged at 1000 g for 2 minutes and supernatant was assayed using the Calcium Liquicolor kit (Stanbio Laboratory, Boerne, TX). CaCl_2_ included in the kit was used to generate a standard curve. The standard curve was used to determine sample calcium concentration based on absorbance values read at 550 nm. Calcium data was normalized to either total protein quantified using the BCA absorbance assay, or to total DNA using the DNA Hoechst fluorescence assay. To visualize calcium accretion, calcium deposits were stained using Alizarin Red S applied directly to the well following a 30-minute fixation in 10% formalin. Images were captured with a Leica (Wetzlar, Germany) EZ 4D Digital Dissecting Scope.

### Endogenous Alkaline Phosphatase

Endogenous alkaline phosphatase (ALP) activity was evaluated at days 10 and 14 of osteogenic differentiation. Alkaline phosphatase enzymatic activity was quantified with the Alkaline Phosphatase Liquicolor® Test (Stanbio Laboratory, Boerne, TX), using the P-nitrophenylphosphate methodology. Briefly, hASC were scraped in RIPA protein lysis buffer and were frozen for storage at −30°C. Samples were then thawed, vortexed and centrifuged (1000 g for 2 min). Supernatant was assayed using absorbance (405 nm) in a kinetic fashion, measuring change in absorbance at 5 minute intervals. Quantified alkaline phosphatase activity was normalized to total protein content using the BCA absorbance assay (570 nm).

### Statistical Analysis

Statistical differences between experimental groups in hASC proliferation, mRNA gene expression, and calcium accretion were analyzed using an unpaired Student's t-test compared to control conditions as denoted on presented graphs. To ensure comprehensive statistical analysis, a one-way, non-repeated measures ANOVA test with a Newman-Keuls multiple comparison test was also used to analyze gene expression and calcium quantification data. A one-way repeated measures ANOVA with a Newman-Keuls post-test was used to analyze alkaline phosphatase kinetic activity data. Statistical significance is indicated as follows: *p<.05, **p<.01, ***p<.001. Data are presented as mean ± standard error of the mean (SEM).

### Controls

hASC transfected for primary cilia protein abrogation were measured against hASC transfected with a non-targeting scramble siRNA knockdown control, and a no knockdown control group of hASC. Cellular controls were cultured under identical conditions in all assays.

## Results

### Identification of Primary Cilia

To initially test our hypothesis, it was necessary to confirm the presence of primary cilia on our hASC populations. Using immunofluorescence to visualize the cilia, a mouse α-acetylated tubulin antibody was used to stain the microtubule cilia structure. Primary cilia were most frequently identified as a dense clustering of acetylated tubulin projecting from the apical surface of the cell membrane **(** **Fig. 1b–f** **)**. Cilia were observed in a variety of conformations within the undifferentiated hASC population after 24 hours **(** **Fig. 1b** **)**, 3 days **(** **Fig. 1c** **)** and 12 days **(** **Fig. 1e** **)** of culture in complete growth medium (CGM), and within the osteogenically differentiated population after 3 days **(** **Fig. 1d** **)** and 12 days **(** **Fig. 1f** **)** of culture in osteogenic differentiation medium (ODM). Cilia length tended to vary with phenotype: cells grown in hASC growth media tended to express shorter cilia **(** **Fig. 1b, 1c, 1e** **)**, while osteogenically stimulated hASC expressed more extended cilia **(** **Fig. 1c, 1f** **)** as they committed to the osteogenic lineage, with the day 12 osteogenically differentiated hASC expressing the longest cilia **(** **Fig. 1f** **)**. A portion of the hASC population consistently expressed primary cilia, however, since their expression is linked to the cell-cycle, primary cilia were observed on approximately 30% of the undifferentiated hASC in sub-confluent culture and up to 60% of hASC in confluent culture (data not shown). Incidence and length of primary cilia on hASC were found to be directly related to the chemical culture environment and cell phenotype.

### siRNA Knockdown of Cilia-Associated Proteins IFT88, PC1, and PC2

**Figure 2:**
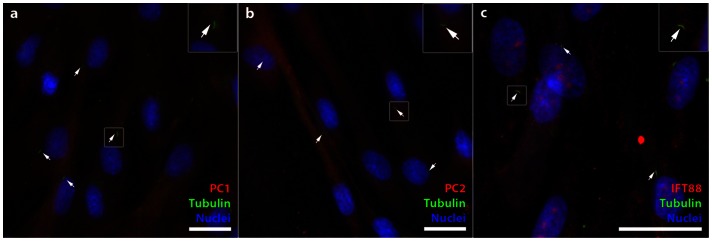
hASC expression of polycystin-1 (PC1), polycystin-2 (PC2) and intraflagellar transport protein-88 (IFT88). (a) PC1 and (b) PC2 are observed as a diffuse signal on the cell body and at the base of the cilium. (c) IFT-88 is localized at the base and tip of the primary cilium. Cilia are identified with anti-acetylated tubulin (green), PC1, PC2 and IFT88 (red), and nuclei are identified with DAPI staining (blue). Scale Bar = 25 µm.

hASC expression of polycystin-1 (PC1) **(** **Fig. 2a** **)**, polycystin-1 (PC2) **(** **Fig. 2b** **)** and intraflagellar protein-88 (IFT88) **(** **Fig. 2c** **)** was determined by immunostaining. To assess cilia co-localization, hASC were also co-immunostained for acetylated α-tubulin. IFT88 colocalized to the base and tip of the ciliary axoneme, as denoted by the arrows **(** **Fig. 2c** **)**. PC1 and PC2 exhibited diffuse expression throughout the hASC cell membrane as well as localization at the base of the cilium **(** **Fig. 2a,b** **)**.

**Figure 3:**
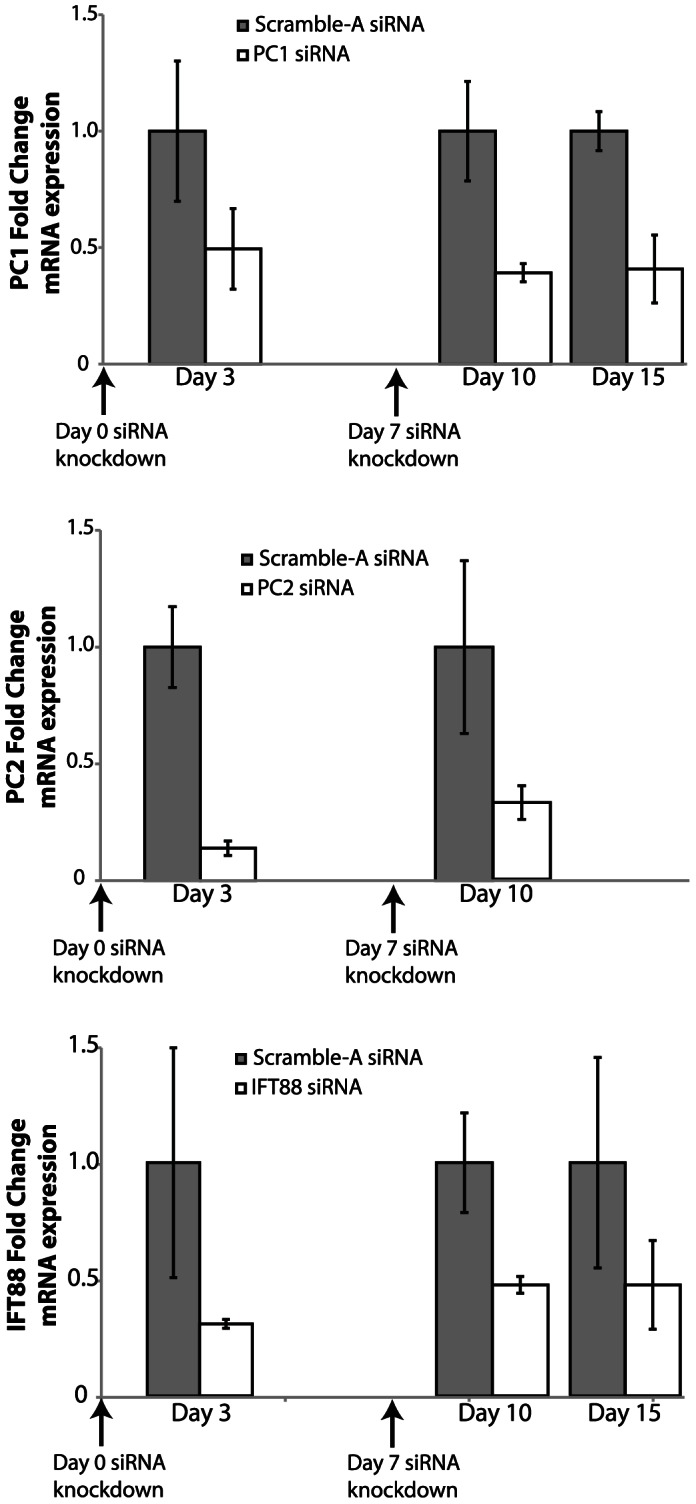
PC1, PC2 and IFT88 gene expression up to 15 days in ODM culture. siRNA knockdown applied at days 0 and 7, confers at least a 50% reduction in mRNA expression of each cilia-associate protein (n = 3, Error bars = SEM).

To elucidate the function of primary cilia-associated proteins in hASC osteogenesis, we systematically abrogated expression of each of the three cilia-associated proteins of interest. To do this, we used serial transient siRNA transfections to allow for sustained mRNA knockdown of PC1, PC2, and IFT88. The serial siRNA knockdown method was used to eliminate any off-target effects associated with other stable knockdown techniques, which can result in a possible alteration in hASC multipotency. This serial siRNA approach yielded a minimum of 50% knockdown efficiency. Transfection with siRNA was performed on day 0 and day 7 of the standard osteogenic differentiation procedure to ensure cilia-associated knockdown for the duration of chemically stimulated osteogenic differentiation. Diminished mRNA expression of PC1, PC2 and IFT88 was confirmed with real-time RT-PCR for up to 15 days of osteogenic differentiation **(** **Fig. 3** **)**.

**Figure 4:**
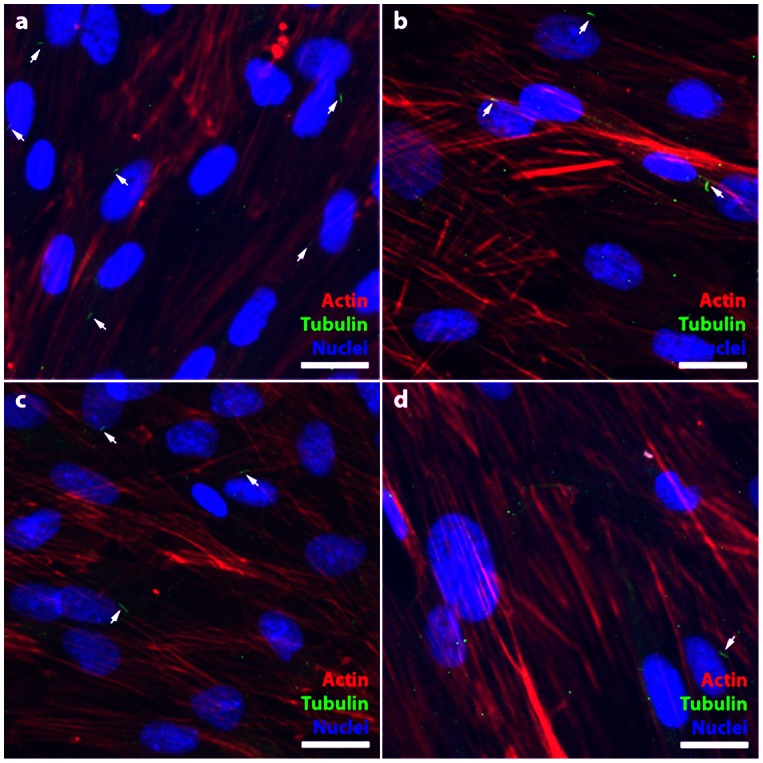
The effects of PC1, PC2 and IFT88 siRNA knockdown on hASC primary cilia. hASC were cultured in expansion media (CGM) for 72 hours. Cilia expression on hASC: (a) Non-targeting scramble siRNA baseline cilia expression on approximately 50% of the hASC population (b) PC1 and (c) PC2 knockdown conditions express more cilia observed in a curved conformation and a minimal decrease in cilia population frequency, (d) IFT88 knockdown confers a reduction in cilia frequency on the hASC population. Cilia are identified with anti-acetylated tubulin (green), actin identified with phalloidin 594 (red) and nuclei are identified with DAPI staining (blue). Scale Bar = 25 µm.

As PC1, PC2 and IFT88 are cilia associated proteins, it is expected that their knockdown would have an effect on cilia structure. hASC were cultured for 72 hours and cilia-associated proteins were assessed via immunostaining **(** **Fig. 4** **)**. We found that IFT88 knockdown had the greatest impact on the frequency of cilia expression observed on the hASC population **(** **Fig. 4d** **)** as compared to the non-targeting scramble siRNA control **(** **Fig. 4a** **)**. Further, we observed that PC1 **(** **Fig. 4b** **)** and PC2 **(** **Fig. 4c** **)** knockdown had a moderate impact on the primary cilia structure with hASC expressing cilia that tended to be expressed in a more curved conformation than cilia in control conditions. PC1 and PC2 knockdown had no noticeable effect on the frequency of cilia observed on the hASC population.

### Cilia-Associated Protein Knockdown Affects hASC Proliferation

**Figure 5:**
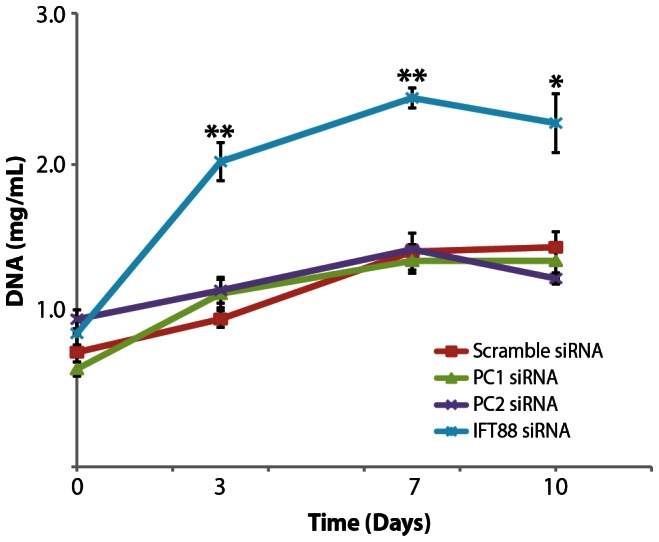
The effect of knockdown on hASC proliferation in expansion media (CGM). DNA was collected at days 0, 3, 7 and 10 to measure proliferative activity with siRNA knockdown of PC1, PC2 and IFT88 in as compared to the non-targeting scramble control siRNA. Knockdown of IFT88 significantly increases hASC proliferation as compared to control (n = 3, error bars = SEM, Student's t-test comparing each treatment to control, * p<0.05, ** p<0.01).

The presence of the primary cilium structure is intimately associated with the cell cycle and thus it was necessary to establish the baseline effects of PC1, PC2, and IFT88 knockdown on the basal hASC phenotype. To assess hASC proliferation activity under siRNA knockdown, the Hoechst DNA Assay was used to measure DNA content incrementally over a 10 day culture in expansion media (CGM) **(** **Fig. 5** **)**. hASC were transfected at day 0 and subsequently cultured in expansion media (CGM) for up to 10 days. We collected DNA at days 0, 3, 7 and 10 and used a fluorimetric assay to measure DNA concentration at each time-point. Interestingly, we found that ITF88 knockdown conferred a significant increase in hASC proliferative activity, whereas PC1 and PC2 knockdown did not alter hASC proliferation as compared to the siRNA non-targeting control **(** **Fig. 5** **)**. The IFT88 knockdown significantly increased hASC proliferative activity as compared to the control. However, PC1 and PC2 exhibited a comparable level of proliferation activity to the control, non-targeting siRNA **(** **Fig. 5** **)**.

hASC are density-dependent; and once they reach confluency, they slow down their proliferative activity and quickly become quiescent. Under phase contrast microscopy, control hASC, PC1- and PC2-knockdown hASC expressed similar cell morphology in confluent culture. In contrast, IFT88 knockdown hASC exhibited a loss of this characteristic hASC density-dependent behavior and rather exhibited crowding and much denser hASC monolayer coverage, as observed by phase contrast microscopy (data not shown).

### Knockdown of Cilia-Associated Proteins Downregulates hASC Osteogenic Gene Marker Expression

**Figure 6:**
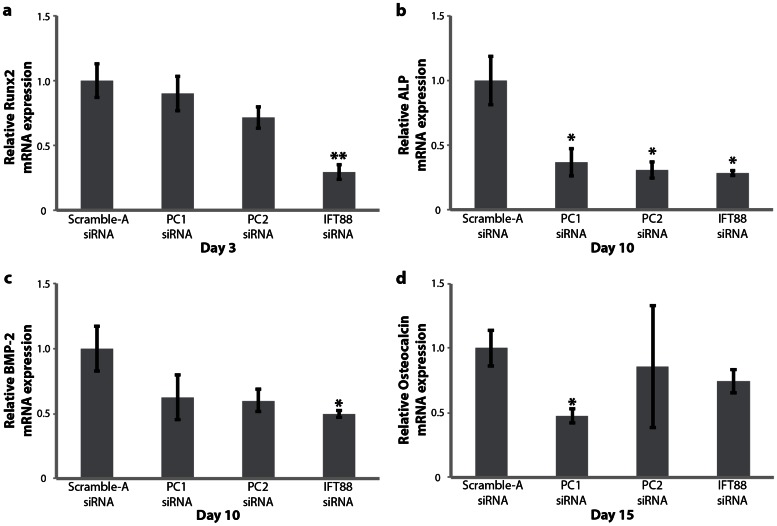
Osteogenic gene marker expression in hASC. IFT88 knockdown confers a reduction in (a) Runx2 gene expression after 3 day and (c) BMP-2 gene expression after 10 day culture in osteogenic differentiation medium (ODM). (b) PC1, PC2 and IFT88 knockdown confer a reduction in alkaline phosphatase (ALP) gene expression after 10 days of culture in ODM. (d) PC1 knockdown confers downregulation of osteocalcin after 15 days of culture in osteogenic differentiation. Knockdown treatments applied at day 0 and day 7 of differentiation, (n = 3, error bars = SEM, Student's t-test comparing each treatment to control, * p<0.05, ** p<0.01).

To evaluate how PC1, PC2 and IFT88 function during the hASC osteogenic differentiation process, real-time RT-PCR was used to analyze mRNA expression for early markers of hASC osteogenesis at days 3,10 and 15 of osteogenic differentiation in ODM. We probed for gene expression of runt-related transcription factor (Runx2) **(** **Fig. 6a** **)** at day 3, gene expression of alkaline phosphatase (ALP) **(** **Fig. 6b** **)** and bone morphogenetic protein-2 (BMP-2) **(** **Fig. 6c** **)** at day 10, and osteocalcin at day 15 **(** **Fig. 6d** **)**, all hallmarks for development of an osteogenic phenotype. After siRNA knockdown and 3 days of culture in osteogenic differentiation medium, IFT88 knockdown resulted in significant downregulation of Runx2 expression, suggesting suppressed osteogenesis **(** **Fig. 6a** **)**. This indicated that the function of IFT88 may be important in the early signals of osteogenic lineage specification in hASC. After siRNA knockdown and 10 days of osteogenic differentiation, we observed that IFT88 knockdown resulted in a significant downregulation of BMP-2 gene expression **(** **Fig. 6c** **)** and PC1, PC2 and IFT88 knockdown resulted in significant downregulation of ALP gene expression **(** **Fig. 6b** **)**. At day 15 of osteogenic differentiation, PC1 knockdown resulted in a significant decrease in osteocalcin expression **(** **Fig. 6d** **)**. Evaluation of osteopontin gene expression was also assessed at day 15 of osteogenic differentiation; however there was no significant change in osteopontin expression with knockdown (data not shown). These results suggest that PC1 and PC2 may function in intermediate signaling processes related to hASC osteogenesis, IFT88 function is necessary at the early-to-intermediary duration of the osteogenic differentiation process, and PC1 in particular is critical in later steps of the osteogenic differentiation process.

### Cell-Mediated Calcium Accretion of Osteogenically Differentiated hASC

**Figure 7:**
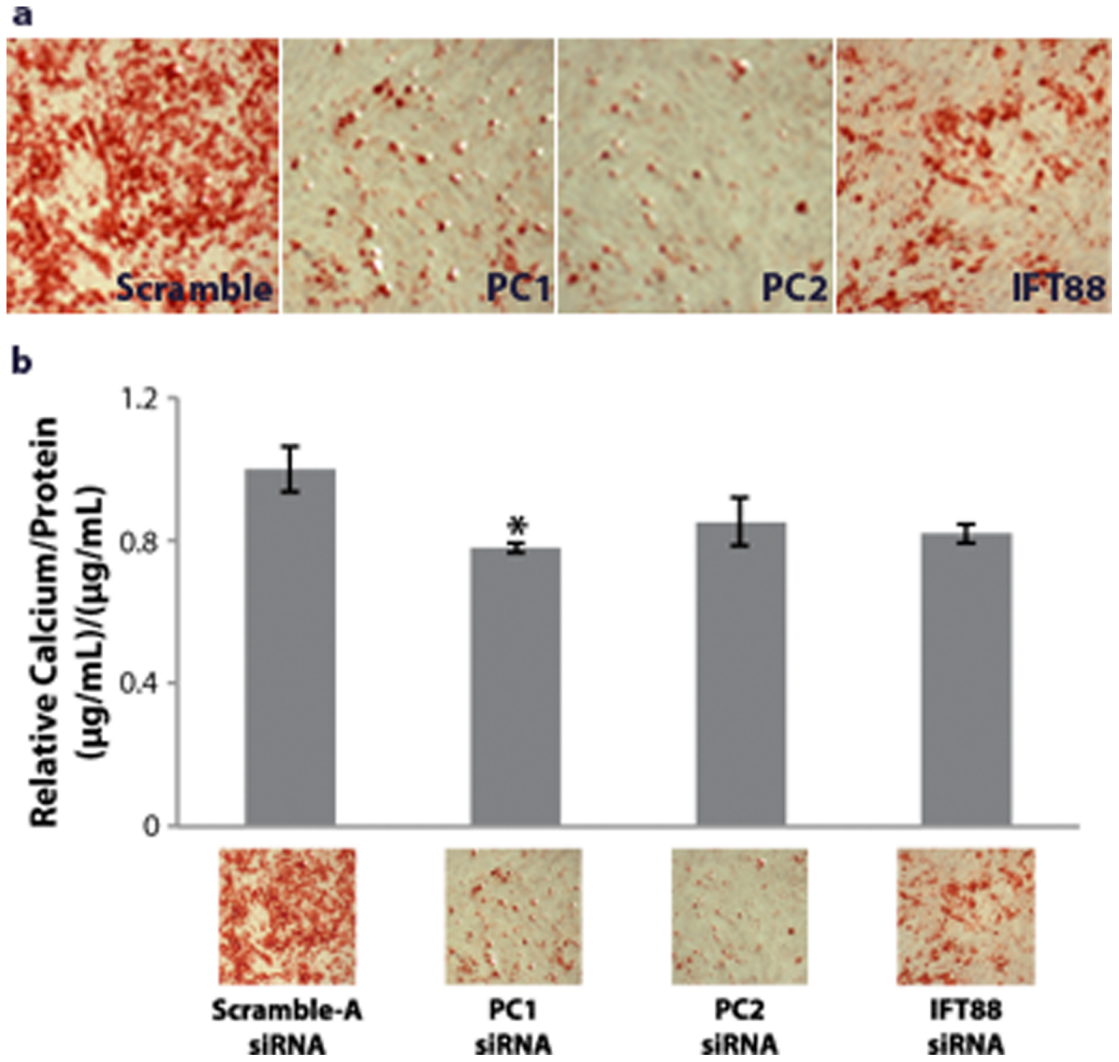
Calcium accretion is affected by cilia-associated protein knockdown. (a) Alizarin Red staining indicating degree of mineralization after 14 days ODM with knockdown. (b) Quantified calcium accretion normalized to DNA content. PC1 and PC2 knockdown leads to diminished calcium accretion, (n = 3, error bars = SEM, Student's t-test comparing each treatment to control, * p<0.05).

Calcium accretion and endogenous alkaline phosphatase activity are canonical hallmarks of functional osteogenesis and development of bone-like calcified tissue. To extend our study further, we looked at the effects of PC1, PC2, and IFT88 knockdown on hASC mediated mineralization after 14 days of osteogenic differentiation to determine if the activity of these proteins was involved in the development of functional osteogenic tissue. Alizarin Red results indicated that PC1 and PC2 knockdown led to a diminished level of calcium accretion after 14 days of osteogenic differentiation with culture in ODM **(** **Fig. 7a** **)**. Quantification of calcium accretion using a colorimetric absorbance calcium assay further corroborated this qualitative data. Evaluating calcium concentration as normalized to DNA content, we observed a diminished calcium concentration **(** **Fig. 7b** **)**. This data suggested that PC1 and PC2 were critical in the ability of hASC to functionally differentiate into an osteogenic mineralized phenotype. Taken together with the gene expression data **(** **Fig. 6** **)**, these findings support the idea that PC1 plays a particular role in the later signaling pathways of hASC osteogenesis.

### PC1, PC2 and IFT88 Affect ALP Enzymatic Activity

**Figure 8:**
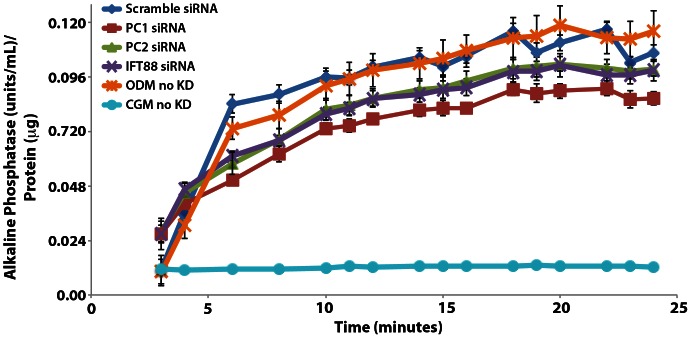
Endogenous alkaline phosphatase (ALP) activity of hASC after 14 days of culture. hASC were cultured in osteogenic differentiation medium (ODM) and transfected with non-targeting scramble, PC1, PC2 and IFT88 siRNA. ODM and complete growth medium (CGM) hASC without knockdown (no KD) were also measured for comparison of baseline activity. Alkaline phosphatase activity is suppressed with cilia-associated protein knockdown. Knockdown of PC1 (*** p<0.01), PC2 (* p<0.05) and IFT88 (* p<0.05) all led to a significant decrease in ALP enzymatic activity compared to the non-targeting scramble siRNA knockdown control (n = 3, error bars = SEM, One-way repeated measures ANOVA, with Newman-Keuls post-hoc test).

End product expression of ALP and its enzymatic activity level is another important hallmark of development of a true osteogenic phenotype. To further understand how PC1, PC2 and IFT88 knockdown suppresses development of a functional osteogenic phenotype in hASC, we evaluated ALP enzymatic activity. At 14 days of culture in ODM, we analyzed ALP activity using a kinetic colorimetric absorbance assay (Stanbio, Boerne, TX) over the course of 25 minutes. Our results indicated that knockdown of PC1, PC2 and IFT88 led to significantly decreased ALP enzymatic activity suggesting diminished development of an osteogenic phenotype **(** **Fig. 8** **)**. This data supports the idea that all three proteins are necessary to induce a hASC osteogenic response, with the greatest suppression of ALP activity observed in the PC1 knockdown **(** **Fig. 8** **)**, consistent with the calcium accretion data **(** **Fig. 7** **)**.

## Discussion

This study has demonstrated that primary cilia are prevalent on hASC and that cilia proteins appear to play a critical role in mediating the osteogenic differentiation of hASC. Additionally, we have determined that the presence of the primary cilium and functional IFT88 is likely linked to early lineage commitment signals and may be important in appropriate hASC proliferation. This proliferative behavior suggests that IFT88 may either play a role in maintenance of a normal baseline hASC progenitor cell phenotype, or it may conversely suggest that IFT88 is necessary to prevent aberrant, over-proliferative activity. A further study analyzing stemness and cell cycle gene markers in IFT88 knockdown hASC will be required to elucidate IFT88 function in progenitor cell types. hASC do not express typical stemness cell markers such as Oct-4, Sox2, and Nanog found in pluripotent stem cells; however they do generally express a panel of consensus cell surface markers distinctive to their multipotent population [8]. Future comparative work characterizing immunophenotypes of wild type hASC and IFT88 knockdown hASC will be critical and interesting mechanistic work in understanding IFT88's role in stemness phenotype. Our findings showed that IFT88 knockdown led to a significant increase in hASC proliferative activity, suggesting the ciliary activity of IFT88 is likely acting in the process of cell cycle progression **(** **Fig. 5** **)**.

Interestingly, IFT88 knockdown of osteogenically differentiated hASC also conferred significant downregulation of Runx2 and BMP-2 gene expression, which are early and intermediary markers for osteogenesis **(** **Fig. 6a, 6c** **)**. The Runx2 result was consistent with previous work in MSC demonstrating IFT88 knockdown disrupted MSC osteogenesis as indicated by diminished Runx2 expression [23]. BMP-2 gene expression is important in the process of wound healing and tissue remodeling in bone and interacts with Hedgehog, Wnt and TGF-β signaling - pathways that localize processes in the primary cilium **[3]**, [24], **[25]**. It follows that IFT88 knockdown disrupts gene expression of BMP-2 as the cilium structure is likely involved in BMP-2 signaling processes, which in turn affect hASC osteogenesis. Taken together these data indicate IFT88 may be an early modulatory switch dictating hASC to operate in either a proliferation signaling modality or the contrasting differentiation signaling modality. These findings hint that the function of IFT88 may be more dynamic than just regulating protein transport in the primary cilium, suggesting instead that it may also participate in the early stages of hASC fate determination. These findings are consistent with IFT88 studies in immortalized cell lines [26].

While IFT88 knockdown confers early effects on hASC differentiation, disrupting early signals modulating osteogenic lineage commitment, PC1 and PC2 knockdown resulted in disruption of later markers of osteogenesis. Both PC1 and PC2 knockdown conferred a downregulation of alkaline phosphatase gene expression **(** **Fig. 6b** **)** and end-product expression conferring a decrease in the endogenous alkaline phosphatase enzymatic activity **(** **Fig. 8** **)**. siRNA knockdown of cilia-specific PC1 and PC2 resulted in reduced calcium accretion **(** **Fig. 7** **)**, which is somewhat intuitive as PC1 and PC2 are thought to couple together forming a calcium channel [14]. However, the PC1–PC2 complex is generally thought to be a stretch activated calcium channel, though the mechanistic function of the PC1–PC2 complex is a matter of debate [14], [27].

In our static culture system, we have observed significant diminished cell-mediated calcium accretion with knockdown of PC1 **(** **Fig. 7b** **)** and downregulation of osteocalcin gene expression at day 15 of differentiation **(** **Fig. 6d** **)**, which suggests that there is also a non-mechanical signaling component in the function of PC1. This finding is consistent with mouse model work showing heterozygous PC1 knockout mice embryos exhibit reduced bone mineralization [28]. Osteocalcin is an osteogenic bone marker molecule, and its gene expression is typically upregulated in osteoblastic cell types [29]. Secreted osteocalcin is known to closely associate with mineralized bone matrix, which supports the critical activity of PC1 in formation of an appropriate bone matrix as evidenced by calcium accretion and osteocalcin gene expression. Our data supports a similar global effect of PC1 knockdown within the static *in vitro* system. Taken together with other recent evidence, our findings suggest that PC1 may have multiple functions in the osteogenic differentiation process and, further, that its function is likely critical to the development of properly mineralized bone.

In general, there was a greater qualitative difference in the alizarin red staining data than the quantitative data for calcium accretion, which may be attributed to variations in cell number, and/or differences in the assays **(** **Fig. 7** **)**. Though IFT88 knockdown did not significantly reduce the amount of calcium accreted, the quantitative trend followed with the staining, showing that knockdown of all three proteins may have an effect **(** **Fig. 7** **)**. This smaller quantitative change may also be attributed to siRNA knockdown efficiency. In a stable knockdown model with complete abrogation of ciliary protein mRNA expression, we would potentially expect a more exaggerated difference between treatments.

Our results diverge somewhat from what is reported in IFT88 knockdown by Pazour *et al.* showing a more severe cilium phenotype in Chlamydomonas and mice than our hASC using transient siRNA techniques [17]. Their study used different model organisms and reported that Chlamydomonas IFT88 mutants did not exhibit an increase in proliferative activity, which is not consistent with the results in our model. Though cilia formation is disrupted in our IFT88 knockdown, it is by no means completely abrogated and this likely contributes to the differences between our findings and those of the Pazour *et al.* study.

Increased endogenous alkaline phosphatase activity is a characteristic marker indicating development of a functional osteogenic phenotype. Knockdown of PC1, PC2 and IFT88 all conferred downregulated alkaline phosphatase gene expression after 10 day exposure to ODM, chemical osteogenic induction **(** **Fig. 6b** **)**. This data was further corroborated with the enzymatic activity of alkaline phosphatase, showing reduced activity in all three knockdown conditions **(** **Fig. 8** **)**. These findings support consistent observations in hASC osteogenesis *in vitro* and evidence of disrupted skeletogenesis in heterozygous mouse embryo knockouts [28].

To summarize our findings, we established the relative temporal activities of PC1, PC2 and IFT88. We generalized that IFT88 activity was critical in earlier stages of hASC lineage specification processes **(** **Fig. 5** **,** **Fig. 6** **)**, whereas PC1 and PC2 activity affected later stages of hASC osteogenic phenotypic development. Osteogenic transcription factor Runx2 and Hedgehog related osteogenic signaling molecule BMP-2 gene expression were sensitive to IFT88 knockdown. PC1 and PC2 activity specifically affects intermediary and later gene expression markers of hASC osteogenesis **(** **Fig. 6** **)**. In particular, PC1 appeared to be critical for later functional hallmarks of the osteogenic phenotype, affecting osteocalcin gene expression **(** **Fig. 6d** **)** and conferring effects at the level of end product expression – calcium accretion and alkaline phosphatase activity **(** **Fig. 7** **,** **Fig. 8** **)**. These data suggest that IFT88 may be a mediator between a proliferative signaling modality **(** **Fig. 5** **)** and a differentiation signaling modality **(** **Fig. 6b,c** **)**, as its significant effects seem to occur at the early stages of differentiation. Further, PC1 is critical in the appropriate expression of end-product expression and the functional activity of osteogenic cells derived from hASC.

This study temporally assayed a subset of typical hallmark osteogenic measures: endogenous alkaline phosphatase activity, upregulation of gene markers, morphological changes, and calcium accretion, but the techniques used in this study were by no means exhaustive. As seen from the mRNA data, each of these specific ciliary protein knockdowns temporally effects alterations in the osteogenic process. The calcium data, taken together with gene expression and alkaline phosphatase activity data suggest that PC1 is more critically involved in the later stages of osteogenesis, which provides a compelling explanation for its effect on calcium accretion. IFT88 is critical in the formation of the ciliary structure and significantly affects early lineage commitment signals. However, the cilium structure is by no means the only mediator of this process. Taken together our data imply that cilia-associated proteins are involved in the osteogenic process, and temporally IFT88 is a mediator of early differentiation signals, while PC1 mediates later lineage commitment signals.

Our results shed light on primary cilia-mediated aspects of hASC osteogenesis. We have established the presence of primary cilia on hASC cell populations and have uncovered primary cilia and its key associated proteins are linked to determining hASC phenotype. Additionally, we have demonstrated that primary cilia play a role in controlling hASC proliferation and thus may also be involved in regulating cell proliferation/cell cycling activity of this cell population. We have established that primary cilia-associated proteins PC1, PC2 and IFT88 all have a chemosensitive role in mediating hASC differentiation and propose that they are critically active proteins in this process, despite their unchanged level of gene expression.

To mechanistically understand PC1, PC2 and IFT88's mode of action, an extensive protein study tracking protein localization, pathway activity and post-translational modifications throughout hASC differentiation should be pursued. While exploring mechanistic cilium mediated signaling is important and interesting, the focus and scope of this study was to initially identify whether the cilium was affecting hASC osteogenesis, and to investigate how ciliary proteins affect typical hallmarks of differentiation in the context of regenerative medicine applications. As the primary cilium is emerging as a critical organelle that modulates a number of signaling and tissue homeostasis properties, we aimed to understand if they were players in hASC osteogenesis. Our ongoing and future work will explore the more intimate mechanisms of cilia modulated hASC differentiation, by means of investigating pathways such as PDGF, Hh and Wnt signaling, known to be important in processes of lineage commitment and localized to the primary cilium. Additionally, a further extension of this work will include incorporation of mechanical stimulation to enhance osteogenesis as all three of these cilia proteins also exhibit mechanosensitivity as chemosensitivity is only part of the story [18], [27], [30].

This work is the first step to understanding the intricacies of primary cilia function in hASC differentiation in order to better harness hASC potential for creating bone tissue replacements. This is the first study to demonstrate that primary cilia play an important role in chemically mediated osteogenic differentiation of hASC.

## Conclusion

We have conclusively demonstrated that primary cilia are present on hASC and that three of their key structural/functional proteins are critical in hASC osteogenic lineage specification. Primary cilium morphology changes with respect to chemically induced changes in cell phenotype. Specifically, we have shown that osteogenically differentiated hASC exhibit longer cilia than hASC cultured in basal growth media. Our study is the first to show that IFT88 is likely an early mediator controlling the switch between a proliferative stem cell phenotype and a committed osteogenic phenotype in hASC. We have also demonstrated that PC1 plays a particularly important role in osteogenic end product expression and is critical in developing truly functional osteogenic-like tissue. Taken together, the results from this study indicate that the structure of the primary cilium is intimately associated with the process of hASC osteogenic differentiation and that its associated proteins are critical players in this process. Elucidating the dynamic role of the primary cilium and its associated proteins will help advance the application of hASC in generating autologous tissue engineered therapies for the potential treatment of critical defect bone injuries.

## References

[1] Singla V (2006) The Primary Cilium as the Cell's Antenna: Signaling at a Sensory Organelle. Science 313: 629–633 doi:10.1126/science.1124534. 16888132 10.1126/science.1124534

[2] Pazour GJ, Witman GB (2003) The vertebrate primary cilium is a sensory organelle. Current Opinion in Cell Biology 15: 105–110 doi:10.1016/S0955-0674(02)00012-1. 12517711 10.1016/s0955-0674(02)00012-1

[3] Santos N, Reiter JF (2008) Building it up and taking it down: The regulation of vertebrate ciliogenesis. Dev Dyn 237: 1972–1981 doi:10.1002/dvdy.21540. 18435467 10.1002/dvdy.21540 PMC3304540

[4] Eggenschwiler JT, Anderson KV (2007) Cilia and Developmental Signaling. Annu Rev Cell Dev Biol 23: 345–373 doi:10.1146/annurev.cellbio.23.090506.123249. 17506691 10.1146/annurev.cellbio.23.090506.123249 PMC2094042

[5] Abou Alaiwi WA, Lo ST, Nauli SM (2009) Primary Cilia: Highly Sophisticated Biological Sensors. Sensors 9: 7003–7020 doi:10.3390/s90907003. 22423203 10.3390/s90907003 PMC3290460

[6] Slough J, Cooney L, Brueckner M (2008) Monocilia in the embryonic mouse heart suggest a direct role for cilia in cardiac morphogenesis. Dev Dyn 237: 2304–2314 doi:10.1002/dvdy.21669. 18729223 10.1002/dvdy.21669 PMC2919240

[7] Goetz SC, Ocbina PJR, Anderson KV (2009) Chapter 10 - The Primary Cilium as a Hedgehog Signal Transduction Machine. First edition. Elsevier. 24 pp. doi:10.1016/S0091-679X(08)94010-3 10.1016/S0091-679X(08)94010-3 PMC2867239 20362092

[8] Gimble JM, Guilak F (2003) Adipose-derived adult stem cells: isolation, characterization, and differentiation potential. Cytotherapy 5: 362–369 doi:10.1080/14653240310003026. 14578098 10.1080/14653240310003026

[9] Zuk PA, Zhu M, Ashjian P, De Ugarte DA, Huang JI, et al (2002) Human adipose tissue is a source of multipotent stem cells. Molecular biology of the cell 13: 4279–4295 doi:10.1091/mbc.E02. 12475952 10.1091/mbc.E02-02-0105 PMC138633

[10] Zuk PA, Zhu M, Mizuno H, Huang J, Futrell JW, et al (2001) Multilineage cells from human adipose tissue: implications for cell-based therapies. Tissue Eng 7: 211–228. 11304456 10.1089/107632701300062859

[11] Bodle JC, Hanson AD, Loboa EG (2011) Adipose-Derived Stem Cells in Functional Bone Tissue Engineering: Lessons from Bone Mechanobiology. Tissue Engineering Part B: Reviews 17: 195–211 doi:10.1089/ten.teb.2010.0738. 21338267 10.1089/ten.teb.2010.0738 PMC3098956

[12] Yoder BK (2007) Role of Primary Cilia in the Pathogenesis of Polycystic Kidney Disease. Journal of the American Society of Nephrology 18: 1381–1388 doi:10.1681/ASN.2006111215. 17429051 10.1681/ASN.2006111215

[13] Satir P, Pedersen LB, Christensen ST (2010) The primary cilium at a glance. Journal of Cell Science 123: 499–503 doi:10.1242/jcs.050377. 20144997 10.1242/jcs.050377 PMC2818190

[14] Zhou J (2009) Polycystins and Primary Cilia: Primers for Cell Cycle Progression. Annu Rev Physiol 71: 83–113 doi:10.1146/annurev.physiol.70.113006.100621. 19572811 10.1146/annurev.physiol.70.113006.100621

[15] Huan Y, Van Adelsberg J (1999) Polycystin-1, the PKD1 gene product, is in a complex containing E-cadherin and the catenins. 104: 1459–1468. 10.1172/JCI5111 PMC481982 10562308

[16] Geng L, Segal Y, Peissel B, Deng N, Pei Y, et al (1996) Identification and localization of polycystin, the PKD1 gene product. 98: 2674. 10.1172/JCI119090 PMC507729 8981910

[17] Pazour GJ, Dickert BL, Vucica Y, Seeley ES, Rosenbaum JL, et al (2000) Chlamydomonas IFT88 and its mouse homologue, polycystic kidney disease gene tg737, are required for assembly of cilia and flagella. J Cell Biol 151 3: 709–18. 11062270 10.1083/jcb.151.3.709 PMC2185580

[18] Malone AM, Anderson CT, Tummala P, Kwon RY, Johnston TR, et al (2007) Primary cilia mediate mechanosensing in bone cells by a calcium-independent mechanism. Proceedings of the National Academy of Sciences 104: 13325–13330 doi:10.1073/pnas.0700636104. 10.1073/pnas.0700636104 PMC1939687 17673554

[19] McCullen S, McQuilling J, Grossfeld R, Lubischer J, Clarke L, et al (2010) Application of low frequency AC electric fields via interdigitated electrodes: effects on cellular viability, cytoplasmic calcium, and osteogenic differentiation of human adipose-derived stem cells. Tissue Engineering Part C: Methods 10.1089/ten.tec.2009.0751 PMC3003917 20367249

[20] Knippenberg M, Helder MN, Zandieh Doulabi B, Semeins CM, Wuisman PIJM, et al (2005) Adipose tissue-derived mesenchymal stem cells acquire bone cell-like responsiveness to fluid shear stress on osteogenic stimulation. Tissue Eng 11: 1780–1788. 16411823 10.1089/ten.2005.11.1780

[21] Hanson AD, Marvel SW, Bernacki SH, Banes AJ, Aalst J, et al (2009) Osteogenic Effects of Rest Inserted and Continuous Cyclic Tensile Strain on hASC Lines with Disparate Osteodifferentiation Capabilities. Ann Biomed Eng 37: 955–965 doi:10.1007/s10439-009-9648-7. 19229619 10.1007/s10439-009-9648-7

[22] Bernacki SH, Wall ME, Loboa EG (2008) Methods in Cell Biology. Elsevier. 22 pp. doi:10.1016/S0091-679X(08)00011-3

[23] Tummala P, Arnsdorf EJ, Jacobs CR (2010) The Role of Primary Cilia in Mesenchymal Stem Cell Differentiation: A Pivotal Switch in Guiding Lineage Commitment. Cel Mol Bioeng 3: 207–212 doi:10.1007/s12195-010-0127-x. 10.1007/s12195-010-0127-x PMC2930791 20823950

[24] Meyer RA, Meyer MH, Phieffer LS, Banks DM (2001) Delayed union of femoral fractures in older rats: decreased gene expression. BMC musculoskeletal disorders 2: 2. 11454240 10.1186/1471-2474-2-2 PMC34552

[25] Rawadi G, Vayssière B, Dunn F, Baron R, Roman Roman S (2003) BMP-2 controls alkaline phosphatase expression and osteoblast mineralization by a Wnt autocrine loop. Journal of Bone and Mineral Research 18: 1842–1853. 14584895 10.1359/jbmr.2003.18.10.1842

[26] Robert A, Margall-Ducos G, Guidotti JE, Bregerie O, Celati C, et al (2007) The intraflagellar transport component IFT88/polaris is a centrosomal protein regulating G1-S transition in non-ciliated cells. Journal of Cell Science 120: 918–918 doi:10.1242/jcs.03422. 10.1242/jcs.03366 17264151

[27] Kottgen M, Buchholz B, Garcia-Gonzalez MA, Kotsis F, Fu X, et al (2008) TRPP2 and TRPV4 form a polymodal sensory channel complex. The Journal of Cell Biology 182: 437–447 doi:10.1083/jcb.200805124. 18695040 10.1083/jcb.200805124 PMC2500130

[28] Xiao ZS, Quarles LD (2010) Role of the polycytin-primary cilia complex in bone development and mechanosensing. Annals of the New York Academy of Sciences 1192: 410–421 doi:10.1111/j.1749-6632.2009.05239.x. 20392267 10.1111/j.1749-6632.2009.05239.x PMC2924156

[29] Raymond MH, Schutte BC, Torner JC, Burns TL, Willing MC (1999) Osteocalcin: genetic and physical mapping of the human gene BGLAP and its potential role in postmenopausal osteoporosis. Genomics 60: 210–217. 10486212 10.1006/geno.1999.5893

[30] Forman JR, Qamar S, Paci E, Sandford RN, Clarke J (2005) The Remarkable Mechanical Strength of Polycystin-1 Supports a Direct Role in Mechanotransduction. Journal of Molecular Biology 349: 861–871 doi:10.1016/j.jmb.2005.04.008. 15894330 10.1016/j.jmb.2005.04.008

